# Phylogeography of influenza A H5N1 clade 2.2.1.1 in Egypt

**DOI:** 10.1186/1471-2164-14-871

**Published:** 2013-12-10

**Authors:** Matthew Scotch, Changjiang Mei, Yilma J Makonnen, Julio Pinto, AbdelHakim Ali, Sally Vegso, Michael Kane, Indra Neil Sarkar, Peter Rabinowitz

**Affiliations:** 1 Department of Biomedical Informatics, Arizona State University, 13212 E Shea Blvd, Samuel C Johnson Bldg, Scottsdale, AZ 85259, USA; 2 Center for Environmental Security, Biodesign Institute and Security & Defense Systems Initiative, Arizona State University, Tempe, AZ, USA; 3 Food and Agriculture Organization of the United Nations (FAO), Cairo, Egypt; 4 Yale Occupational and Environmental Medicine Program, Yale University, New Haven, CT, USA; 5 Department of Biostatistics, Yale School of Public health, Yale University, New Haven, CT, USA; 6 Center for Clinical and Translational Science, Department of Microbiology & Molecular Genetics, University of Vermont, Burlington, VT, USA; 7 Department of Computer Science, University of Vermont, Burlington, VT, USA

**Keywords:** Influenza a virus, H5N1 subtype, Phylogeography, Egypt, Epidemiology, Zoonoses

## Abstract

**Background:**

Influenza A H5N1 has killed millions of birds and raises serious public health concern because of its potential to spread to humans and cause a global pandemic. While the early focus was in Asia, recent evidence suggests that Egypt is a new epicenter for the disease. This includes characterization of a variant clade 2.2.1.1, which has been found almost exclusively in Egypt.

We analyzed 226 HA and 92 NA sequences with an emphasis on the H5N1 2.2.1.1 strains in Egypt using a Bayesian discrete phylogeography approach. This allowed modeling of virus dispersion between Egyptian governorates including the most likely origin.

**Results:**

Phylogeography models of hemagglutinin (HA) and neuraminidase (NA) suggest Ash Sharqiyah as the origin of virus spread, however the support is weak based on Kullback–Leibler values of 0.09 for HA and 0.01 for NA. Association Index (AI) values and Parsimony Scores (PS) were significant (p-value < 0.05), indicating that dispersion of H5N1 in Egypt was geographically structured. In addition, the *Ash Sharqiyah* to *Al Gharbiyah* and *Al Fayyum* to *Al Qalyubiyah* routes had the strongest statistical support.

**Conclusion:**

We found that the majority of routes with strong statistical support were in the heavily populated Delta region. In particular, the Al Qalyubiyah governorate appears to represent a popular location for virus transition as it represented a large portion of branches in both trees. However, there remains uncertainty about virus dispersion to and from this location and thus more research needs to be conducted in order to examine this.

Phylogeography can highlight the drivers of H5N1 emergence and spread. This knowledge can be used to target public health efforts to reduce morbidity and mortality. For Egypt, future work should focus on using data about vaccination and live bird markets in phylogeography models to study their impact on H5N1 diffusion within the country.

## Background

Highly pathogenic avian influenza (HPAI) H5N1 is a subtype of influenza A virus that has killed millions of birds and raises serious public health concern because of its potential to spread to humans and cause a global pandemic. Since the original human cases of H5N1 originated in Asia [1], much of the attention concerning the virus has been in Asian countries. However, countries farther to the west such as Egypt have also been impacted by the disease and offer important clues about zoonotic transmission between animals and humans. As of June 2013, the country has reported 173 confirmed human cases to the World Health Organization (WHO) [2], the highest number outside of Asia. Of these cases, 63 have died, equaling a case fatality rate of 36%. Since 2005, Egypt has also reported thousands of avian cases [3]. These numbers have led many experts to consider Egypt as a new epicenter for the disease [4]. Epidemiology studies have suggested that the most common scenario for human infection is close contact with domestic poultry [5-8] such as exposure through live bird markets (LBMs) [9].

Phylogeography is a field that focuses on the geographical lineages of species such as vertebrates or viruses [10]. This science relies on sequence data and geographical information including location of a mortality event or a trapping site. There has been a growing interest in phylogeography of zoonotic RNA viruses [11-13] because of their often shorter genomes and rapid evolution compared to other infectious agents [11]. This science has been used to explore the evolutionary history of virus spread, including different subtypes of influenza.

Early work on the viral sequences of Egyptian H5N1 used bioinformatics to model molecular phylogenetic evolution but did not consider geographic dispersion between different regions within the country [14,15]. Phylogenetic analysis indicated a distinct clade of European-Middle Eastern-African (EMA) isolates [15] that is also classified as clade 2.2 [16]. EMA had three sub-clades with genetic similarities despite being geographically distinct [15].

While global analysis indicated that Egyptian strains were different than many of the early Asian cases, studies also illustrated its relationship to countries that are much closer in geographic proximity including those in Africa [17,18]. In particular, analysis by Cattoli *et al.*[18] indicated that the Egyptian strains formed a monophyletic cluster across all gene segments. This suggested limited nucleotide diversity and that the isolates originated from the same ancestor.

### Geographic analysis of molecular evolution in Egypt

One of the first papers to focus on geographic distribution of the initial Egyptian isolates was done by Aly *et al.*[19]. As found in [15], the results suggest that the Egyptian strains clustered together with other African and Middle Eastern [19] strains. Geographically, the disease was spread over the country’s four main areas: Cairo, Nile Delta, Canal, and Upper Egypt. The Nile Delta region had the highest total, which is consistent with other work [4] and represents about half of the country’s population.

Additional phylogenetic analysis [4,16,20] suggested co-circulation between Egyptian isolates collected in 2008 and isolates from 2006 and 2007. This work highlights the diversity of HPAI H5N1 over a limited time frame. This diversity could be to viral adaptation in response to a country-wide vaccination program [20]. Balish *et al.*[21] expanded on this work using cases from 2007 and 2008. The authors performed phylogenetic analysis of HA sequences and found that almost all viruses within clade 2.2.1 to be from Egypt with five sub-groups within 2.2.1 having strong bootstrap support [21]. In addition, the authors identified certain 2007 and 2008 isolates from northern Egypt to be distinct from 2.2.1 [21], confirming the work by Abdel-Monheim *et al*. [20] that co-circulation of H5N1 was present in the country. The authors noted some geographic variation with groups A and B primarily from southern governorates [21]. However, the mixing of Nile Delta region across all of the groups [21] indicated that vicariance was not constant.

More recent work by Eladl *et al.* described genetic characterization of HPAI H5N1 from Egyptian poultry farms during the 2006–2009 time period and classified three main groups within clade 2.2.1- A, B, and C [22]. Related to this work, the World Health Organization (WHO)-World Organization for Animal Health (OIE)-Food and Agriculture Organization (FAO) working group recently updated H5N1 nomenclature and included the clade 2.2.1.1 within the Egyptian strains to represent these new variants [23]. Arafa *et al.*[24] compared 2.2.1 strains to the variant strains of 2.2.1.1. The authors reported that 2.2.1.1 was found more in commercial poultry that were vaccinated. Conversely, 2.2.1 was found to be linked more to poultry in backyard farms [24].

### Need for phylogeography

By considering geography as a state within genotype evolution, it is possible to infer locations that are impacted by certain clades. This also enables epidemiologists to understand the migration from a given origin to a perceived endpoint; information that is vital for controlling spread of disease. Pinpointing specific trade routes leading to virus propagation within the country can enable targeted inspections and other disease control measures, improving the efficient use of public health resources.

In this paper, we analyze the phylogeography of H5N1 in Egypt and focus on the variant clade 2.2.1.1. The results will highlight the usefulness of phylogeography for public health surveillance and the analysis of disease transmission within the country. In addition, we discuss implications for future spread of the disease between animals and humans in Egypt. The current endemic situation continues to be a major challenge to the poultry industry and regulatory authorities in Egypt. Understanding the epidemiology of the disease is a key element in HPAI control. It is essential to link this knowledge with action to effectively limit the spread of the virus. An understanding of the “usual” patterns of geo-movements of the disease leads to a better understanding of disease pathways and spread. This in turn allows for planning strategies to reduce risks, set priorities, allocate resources effectively and efficiently, and achieve higher benefit-cost ratios with existing or minimal resources [25].

## Results and discussion

**Figure 1:**
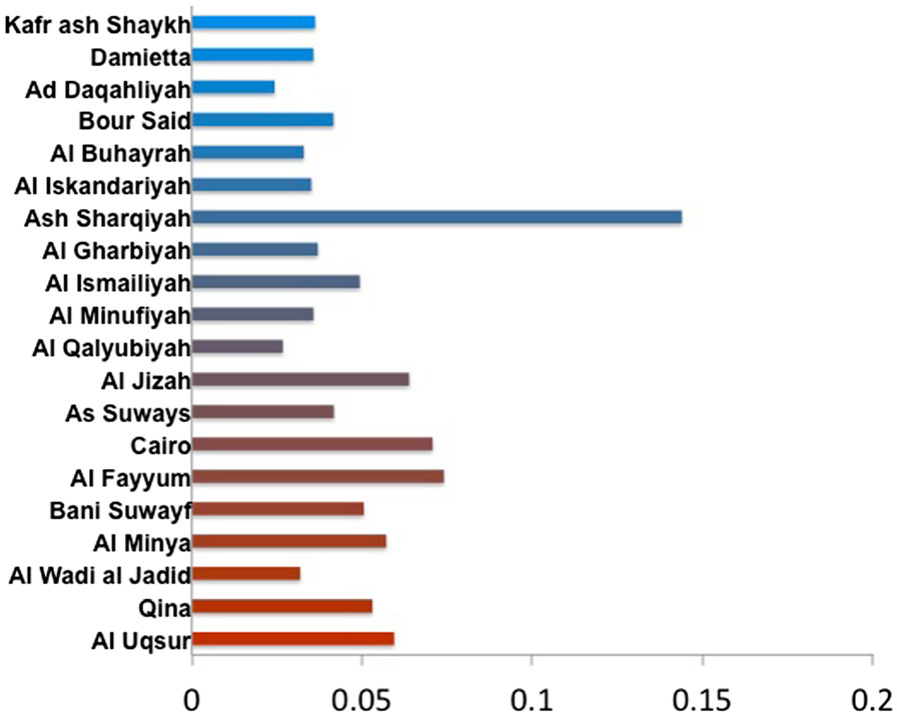
**HA root state posterior probability.** Here Ash Sharqiyah in indicated as the most likely origin of H5N1 in Egypt. The bar graph is colored by gradient from blue for the northern governorates to red for the southern governorates.

**Figure 2:**
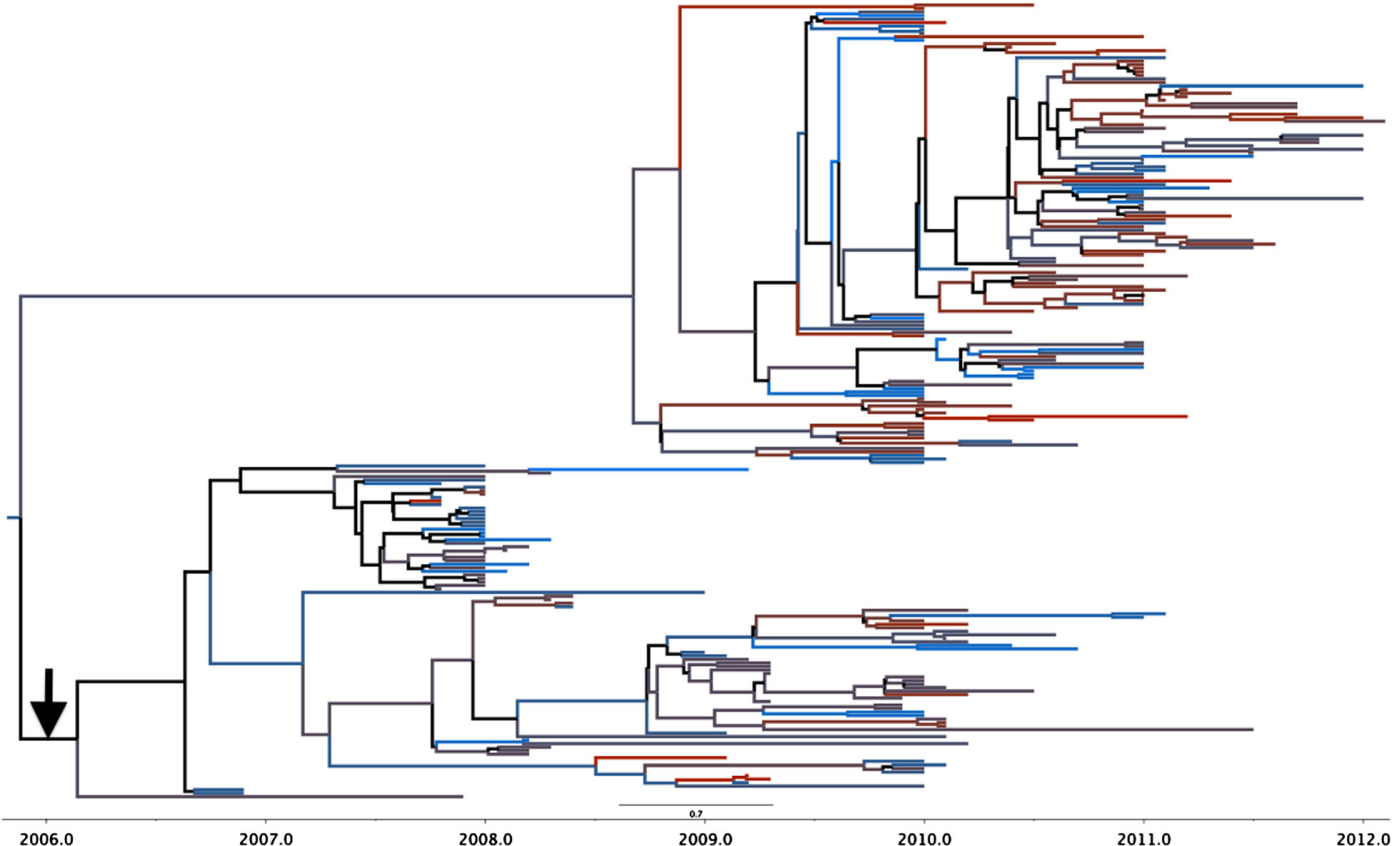
**MCC tree for HA gene.** The color of the branches correspond to the governorates in Figure 1. Black branches in the tree indicate weak support (posterior probability < .65). The black arrow indicates clade 2.2.1.1.

Figures 1 and 2 shows the HA root state posterior probability along with the time-scaled Maximum Clade Credibility (MCC) tree. In Figure 1, the governorates are assigned a color in a gradient from blue to red. Blue represents the northern Delta region, while red signifies Cairo and the land to the south. These colors match the branches in the MCC tree in Figure 2. The Ash Sharqiyah governorate is weakly supported as the most likely site of origin with a probability of 0.14. In addition, the Al Qalyubiyah governorate appears to contain the majority of the routes (branches) in the tree. However, it is connected to black branches that signify a posterior probability less than 0.65. This suggests uncertainty in H5N1 transition to and from Al Qalyubiyah. The 2.2.1.1 sequences formed their own distinct clade and are indicated by the arrow. This clade also includes 21 of the 152 2010–2012 sequences (14%) added from the IRD database.

**Figure 3:**
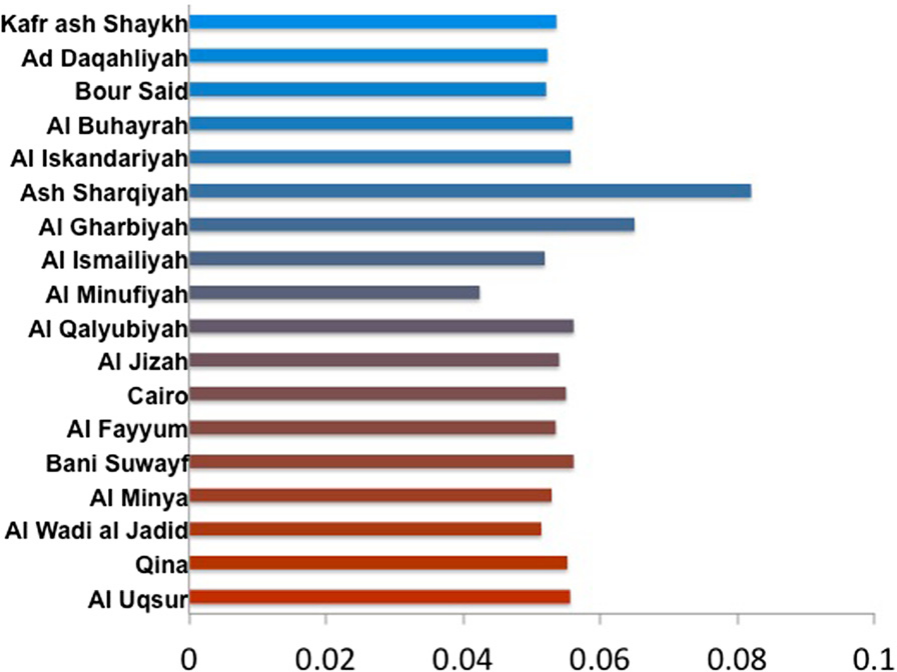
**NA root state posterior probability.** Like the HA results, Ash Sharqiyah in indicated as the most likely origin of H5N1 in Egypt. The blue to red color gradient represents northern to southern governorates.

**Figure 4:**
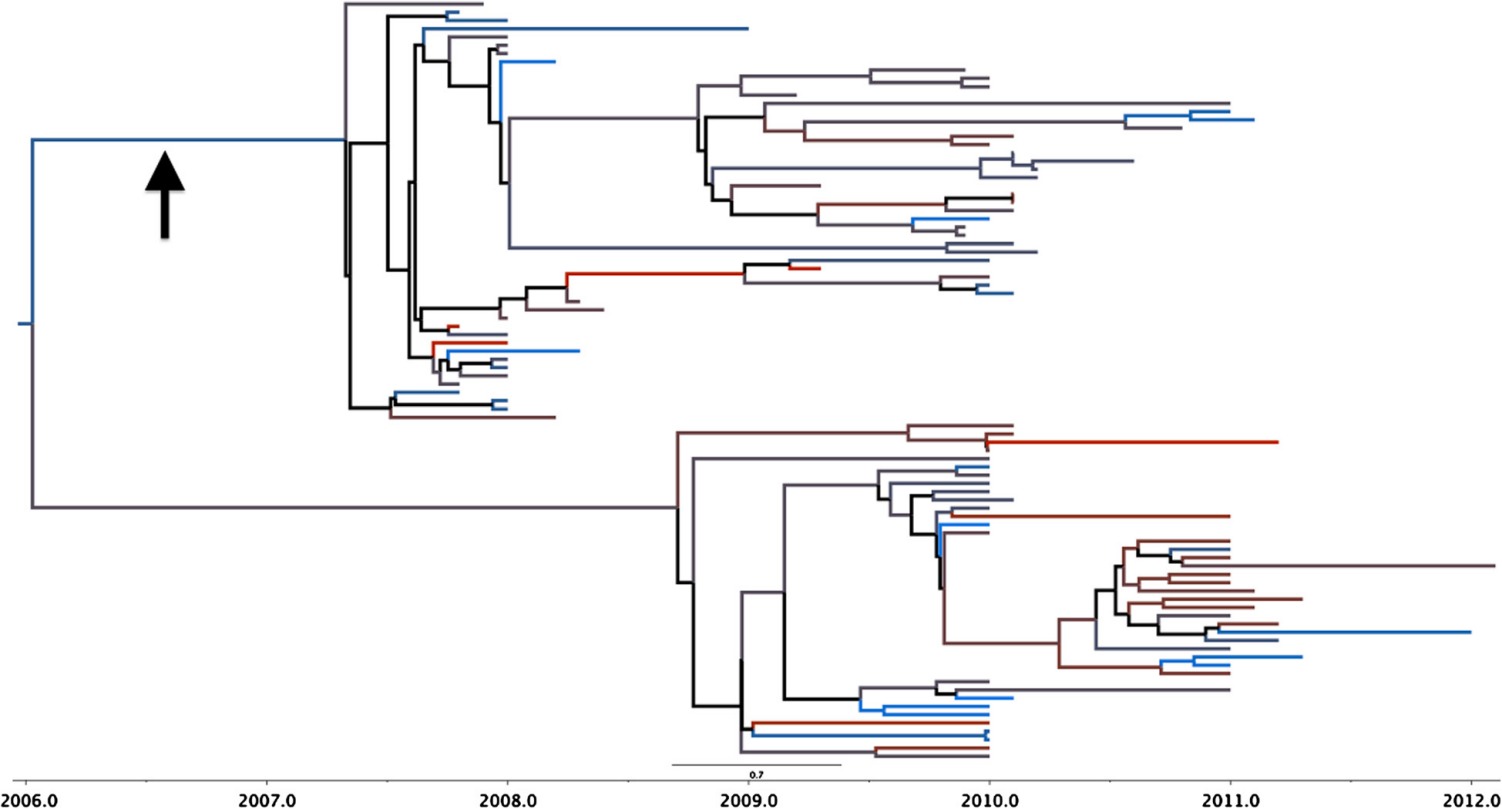
**MCC tree for NA gene.** The color of the branches correspond to the governorates in Figure 3. Black branches in the tree indicate weak support (posterior probability < .65). The black arrow indicates clade 2.2.1.1.

Figure 3 shows the values for the root state in the NA model and indicates Ash Sharqiyah as the origin with a 0.08 posterior probability. Like the HA tree, the Al Qalyubiyah governorate appears to contain the majority of the branches and the 2.2.1.1 sequences form their own distinct clade (Figure 4). This clade also includes sixteen of the 57 2010–2012 sequences (28%) added from the IRD database.

Table 1 shows additional statistical phylogeography metrics including the Kullback–Leibler test that estimates divergence of prior and posterior probabilities of the root state. Here, we used a fixed prior for each tree *1/K*, where *K* is the number of unique states, and the posterior estimates reported in Figures 1 and 3. The small numbers indicate that the phylogeographic models are not able to generate root state posteriors that are vastly different from the underlying priors and thus achieve a low statistical power [13]. In addition, the observed Association Index (AI) and Parsimony Scores (PS) are significant indicating that the evolutionary diffusion of H5N1 in Egypt is geographically structured. These metrics produced p-values that were statistically significant at the 0.05 level.

**Table 1:** Statistical phylogeography metrics

**Gene**	**KL**	**Association index**		**Parsimony score**	
		**Observed**	**Expected**	**Observed**	**Expected**
HA	0.09	12.62	22.25	113.15	162.03
		(11.46 – 13.80)*	(21.35 – 23.23)	(109.00 – 117.00)*	(157.43 – 167.39)
NA	0.01	6.21	9.45	49.18	65.22
		(5.42 – 6.99)*	(8.65 – 10.10)	(47.00 – 52.00)	(62.50 – 67.90)

A higher Kullback–Leibler (KL) value indicates more divergence of the root state posterior probability from its prior [13]. Also shown is the Association Index (AI) and the Parsimony Score (PS) from BaTS. For AI and PS, statistical significance indicates the diffusion is structured by geography [49]. Both HA and NA had an AI and PS p-value < 0.05.

^*^p-value < 0.05.

**Figure 5:**
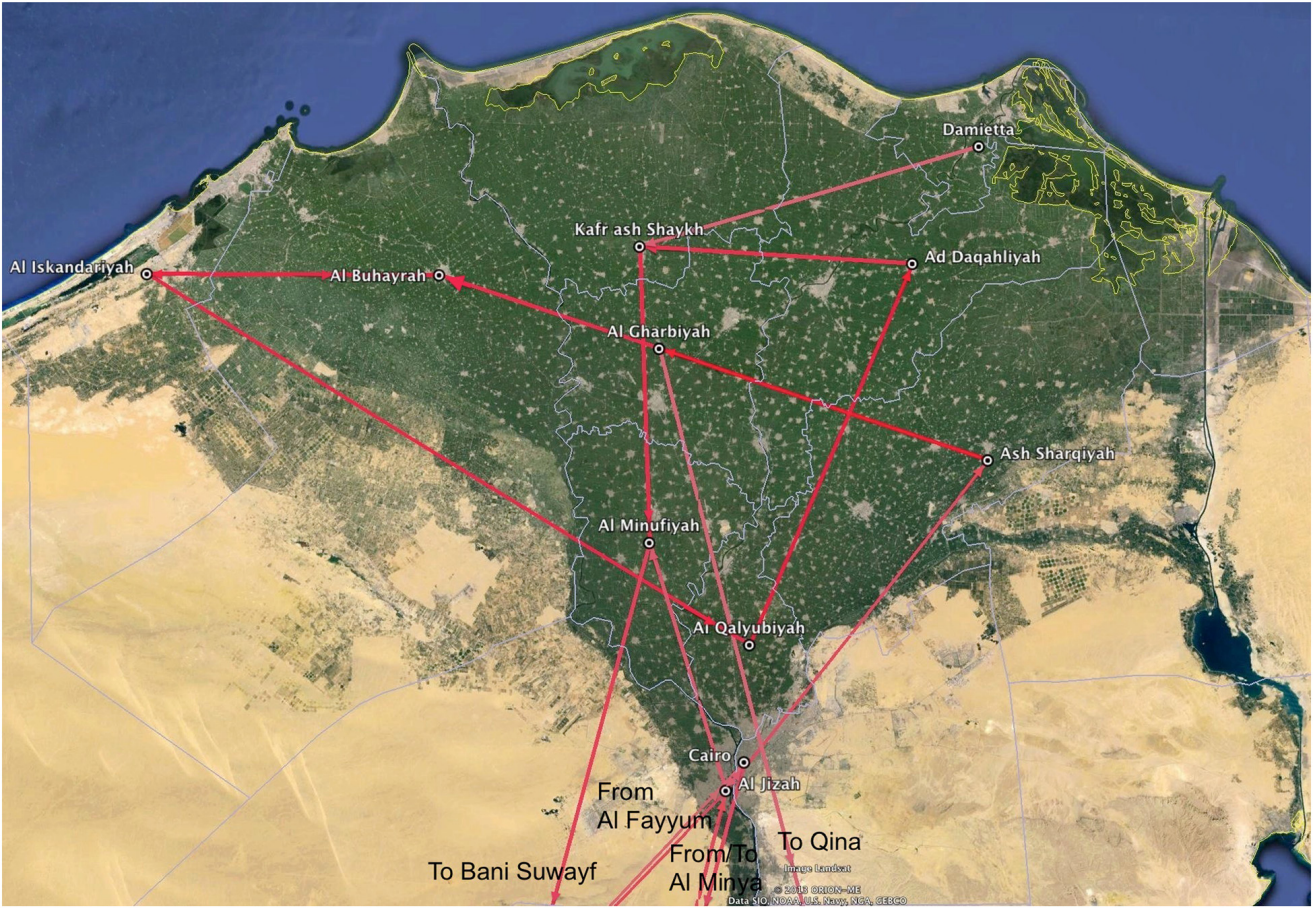
**Bayes factor test for non zero rates for the HA gene.** The software Spread [45] was used to calculate the Bayes Factor for the migration routes between Egyptian cities. A Bayes Factor cutoff of 6 was used. A darker color indicates a higher Bayes Factor. The route with the highest value was Ash Sharqiyah → Al Gharbiyah with a Bayes Factor of 92.86. The map is a zoom-in of the Delta Region.

**Figure 6:**
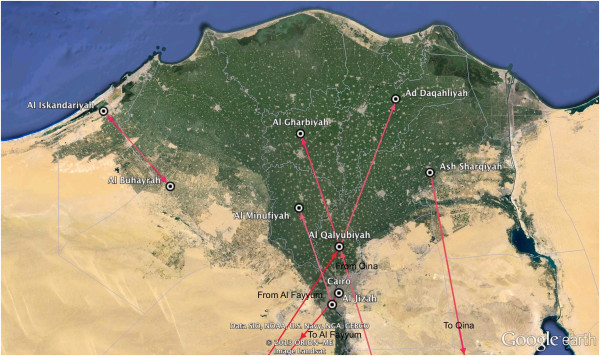
**Bayes factor test for non zero rates for the NA gene.** A Bayes Factor cutoff of 6 was used. A darker color indicates a higher Bayes Factor. Here, the Al Fayyum → Al Qalyubiyah route had the strongest support with a Bayes Factor value of 34.40. The map is a zoom-in of the Delta Region.

Figures 5 and 6 show maps of the HA and NA routes respectively when calculating a Bayes Factor (BF) for the most significant non-zero rates. We used a BF cutoff of six to determine significance (Table 2). In Figure 5, 19 significant HA routes were found. Most of the routes remained in the Delta region, suggesting that it played an important role in virus migration. The route with the strongest support was Ash Sharqiyah → Al Gharbiyah with a BF of 92.86.

**Table 2:** Bayes factor test for non zero rates for both genes

**Gene**	**BF**	**From**	**To**
HA	92.86	Ash Sharqiyah	Al Gharbiyah
HA	56.70	Bani Suwayf	Al Fayyum
HA	48.04	Al Qalyubiyah	Ad Daqahliyah
HA	38.69	Al Buhayrah	Al Iskandariyah
HA	32.35	Al Iskandariyah	Al Qalyubiyah
HA	29.13	Al Gharbiyah	Al Buhayrah
HA	28.51	Kafr ash Shaykh	Al Minufiyah
HA	26.28	Ad Daqahliyah	Kafr ash Shaykh
HA	17.00	Al Minufiyah	Bani Suwayf
HA	14.06	Cairo	Al Minya
HA	14.00	Al Fayyum	Al Jizah
HA	12.94	Al Jizah	Ash Sharqiyah
HA	12.56	Al Minya	Al Jizah
HA	8.99	Al Jizah	Al Minufiyah
HA	6.81	Al Jizah	Cairo
HA	6.60	Damietta	Kafr ash Shaykh
HA	6.31	Cairo	Al Jizah
HA	6.25	Al Gharbiyah	Qina
HA	6.02	Al Fayyum	Cairo
NA	34.40	Al Fayyum	Al Qalyubiyah
NA	27.56	Cairo	Al Fayyum
NA	11.77	Al Iskandariyah	Al Buhayrah
NA	10.61	Al Qalyubiyah	Al Gharbiyah
NA	10.58	Ash Sharqiyah	Qina
NA	9.18	Al Buhayrah	Al Iskandariyah
NA	7.83	Al Qalyubiyah	Ad Daqahliyah
NA	6.85	Qina	Al Qalyubiyah
NA	6.04	Al Jizah	Al Minufiyah

Significant routes had a Bayes Factor (BF) of at least 6.

For the NA tree, nine routes were identified with the Al Fayyum → Al Qalyubiyah route having the highest support with a BF of 34.40. Like the HA, the majority of the routes begin and end in the Delta region. However, it also contains a route from the Delta region in the north to the Qina governorate in the south. This is consistent with the HA map.

We conducted a phylogeography study of H5N1 that focused on Egyptian strains of the variant clade 2.2.1.1. The utilization of viral sequence data and generation of phylogeographic models highlighted the complex history of the virus and the countrywide spread during a small time period. As mentioned, phylogeography allows one to infer geographic dispersion as a component of genotype evolution over time. By considering geography as a *state* within H5N1 evolution, we identified locations that are impacted by certain clades; in this case the variant 2.2.1.1. Our findings are consistent with Arafa *et al.*[24], Balish *et al.*[21] and Taha *et al.*[26], in identifying the Delta region as an important area for 2.2.1.1. In addition, Al Qalyubiyah, also in the Delta, dominated a large portion of both trees, yet there was uncertainty about migration to and from this location. Finally, the statistical phylogeography metrics suggest that H5N1 diffusion is geographically structured in Egypt.

### Agricultural practices in Egypt

In Egypt, the frequent movement of birds, fomites, and traders between different markets and farms provides potential for rapid geographic dispersion of influenza viruses [27]. Al Qalyubiyah and Ash Sharqiyah are governorates characterized by high poultry flock densities with a mean value of more than 8 flocks/km^2^[28]. About 80% of broiler farms apply some degree of vaccination protocol [29] which limits the probability of virus detection. Meanwhile, Cairo is generally considered as the biggest market for live birds in the country and for importation of birds from Delta governorates. LBMs and poultry shops are important sources for the purchase and trading of birds in Cairo and other parts of Egypt. Due to a cultural preference to consume freshly slaughtered poultry, LBMs and poultry shops absorb about 80% of the total commercially produced poultry in the country [30]. LBMs, which are considered to be a continuing source of influenza because of the dense concentration and high rate of live bird turn-over, provide ample conditions for virus amplification and may therefore be important reservoirs for HPAI strains from neighboring governorates and “hubs” of circulation [31]. Al Qalyubiyah alone produces about 69 percent of the native *Baladi* chicken and is considered a center for production and trading of fertile *Baladi* eggs that are often taken and hatched in southern governorates such as Sohag. Southern governorates like Al Uqsur purchase either day-old birds such as Peking ducklings and *Baladi* chicks or fertile eggs from Delta governorates of Al Qalyubiyah, Ash Sharqiyah and Gharbiyah [32]. Sharqiyah is the main source for fertile eggs and day-old Peking and *Baladi* birds for other governorates. Thus, Cairo’s LBMs are linked to Al Qalyubiyah and Ash Sharqiyah farms that are also the main sources of fertile eggs and day old birds for southern governorates.

The authors recognize several limitations with this work including the use of governorate-level geography to infer geographic dispersion. We utilized the centroid latitude and longitude for each governorate and this likely does not reflect the true location of each host that was represented in the sequences. Our previous work highlights the lack of sufficient geographical metadata in GenBank and the need for biomedical informatics approaches to enhance the quality of this key element of phylogeography [33].

Our phylogeographic models found several similarities and differences between the HA and NA datasets. Both models indicated weak support for Ash Sharqiyah as the origin of the spread. However, there were differences about significant dispersion routes. These discrepancies are potentially the result of reassortment in the influenza genome, differences in the size of the two data sets, or due to the Bayesian phylogeographic models themselves. Related to this, another limitation is that we did not compare other approaches of molecular evolution including maximum likelihood and maximum parsimony. Our purely discrete model has limitations including inferring the migration paths by only considering the observed locations. For example, removing a state that was observed only once in both data sets would remove it completely from the model and would thus not be considered in the migration history. Hence, these findings are based on a closed system of locales and genetic samples and may not provide a complete representation of influenza diffusion within Egypt.

## Conclusions

The purpose of this work was to study the phylogeography and spread of H5N1 in Egypt. We examined the variant clade 2.2.1.1 using HA and NA sequences. We analyzed one model for each and both indicated Ash Sharqiyah as the origin of the spread although the statistical support was weak. We also identified the routes between governorates that had strong statistical support. The majority of these were found in the heavily populated Delta region. In particular, the Al Qalyubiyah governorate appears to represent a popular location for virus transition as it represented a large portion of branches in both trees. However, there remains uncertainty about virus dispersion to and from this location and thus more research needs to be conducted in order to examine this.

Phylogeography of H5N1 HPAI in Egypt can enhance health experts’ understanding of viral diffusion between governorates and the characteristics between established and emergent clades. Results from this and similar analyses can be used to target interventions and infection control measures to reduce pathogen transmission. However, future work should focus on using data about vaccination and live bird markets in phylogeography models to study their impact on H5N1 diffusion within the country.

## Methods

We used the WHO-OIE-FAO working group’s neighbor joining phylogenetic tree of 2,947 H5N1 hemagglutinin (HA) strains to identify those classified in clade 2.2.1.1 [34]. In total, 91 sequences were included in this category. One was not available in GenBank and thus the remaining 90 were downloaded from GenBank in FASTA format. We provided metadata for each sequence and included the year of collection, the type of host, and the location of the host. We identified the metadata by analyzing the strain name of the sequence such as A/chicken/Egypt/1/2008. Since location metadata is essential for phylogeography our inclusion criterion was for each sequence to have a location metadata value at the governorate level. Most of the strain names in our data set only had *Egypt*, thus we analyzed the *country* metadata field in each GenBank record for more specific information. In total, 74 (82%) HA sequences had a governorate name in the country metadata field and were included in the final data set. For each governorate name, we identified the latitude and longitude of its centroid location. We used the centroid for each governorate due to the absence of more granular locations such as cities. For governorates with vast surface areas, this creates large uncertainty in regards to the true location. However, our focus was on the diffusion of influenza between governorates and the use of centroid locations enables for a consistent approach. Finally, the most recent date in clade 2.2.1.1 [34] was only *2010*, thus we used the Influenza Research Database (IRD) [35] to downloaded additional HA sequences from 2010–2012 in Egypt. For the HA gene, IRD considers a complete segment to contain at minimum 1,659 sequences. Thus, we specified this minimum segment length and also excluded sequences without a governorate name. This added 152 sequences to the final set for a total of 226.

We could not identify a separate tree for the NA strains, thus we included any strains in the HA tree that also had a NA sequence available in GenBank. In total, 37 sequences were downloaded from GenBank. 35 NA sequences (95%) had a governorate name in the metadata field and were included in the final dataset. We again used the IRD to identify additional NA sequences from Egypt from 2010–2012. This added 57 sequences to the final set for a total of 92. However, due to the low total, we did not specify a minimum segment length. Additional files 1 and 2 show maps of the Egyptian governorates with the number of HA and NA sequences (respectively) included in this study.

### Phylogeography

We modeled our approach after the work done by Lemey *et al.* for studying the phylogeography of H5N1 on a global perspective [13]. We used ZooPhy [36], a system developed by one of the authors (MS) for performing phylogeography on zoonotic viruses. ZooPhy contains a workflow of webservices that includes: sequence alignment via ClustalW [37,38], testing of DNA substitution models via jModeltest [39,40], creation of Bayesian phylogeographic trees via BEAST [13,41], and selection of the maximum clade credibility (MCC) tree via TreeAnnotator [41]. For some of the models, we used Saguaro, a high-performance supercomputer at Arizona State University [42] to run BEAST 1.6.

We considered multiple scenarios in our Bayesian discrete model. This includes the use of a strict or relaxed molecular clock, and a reversible or non-reversible phylogeographic diffusion between states. Thus, we evaluated four separate scenarios for each gene: strict-reversible, strict-nonreversible, relaxed-reversible, and relaxed-nonreversible. For the relaxed model, we used an uncorrelated log-normal scenario. We set the length of the Bayesian Markov chain Monte Carlo (MCMC) run to 20 million for NA and 40 million for the larger HA dataset; sampling every 1,000 steps. We compared models by estimating the log marginal likelihood values via the Bayes Factor test using the Tracer program [43]. For both HA and NA, the relaxed-nonreversible model was the best and thus was used for the analysis. Regarding nucleotide substitution models, jModeltest selected TIM1 + G for HA and GTR + G for NA.

For the NA dataset, we re-ran an additional MCMC of 20 million steps and then performed a series of extra steps offline. First, we compared the two log files for each gene using Tracer and found them to be congruent. We then used LogCombiner [44] to combine the log files and tree files to increase our sample size for both genes. We created the MCC trees by using TreeAnnotator and specifying a 10% burn-in and a 0.65 posterior probability limit. For the HA dataset, the effective sample size (ESS) values were low for certain operators after 40 million steps. We decided to increase the chain length to 100 million and then combine the two models in order to increase the ESS values.

### Statistical phylogeography

We also estimated the number of non-zero rates of dispersion between discrete location states. We used this to calculate the Bayes Factor which estimates the routes in the phylogeographic model with the strongest support [13]. Since we used a nonreversible model, we can infer directionality for a given route such as A → B or B → A. The Bayes Factor was calculated for both the HA and NA sequences using SPREAD [45], a separate software application which also generates a keyhole markup language (KML) file for viewing in Google Earth.

We calculated three additional metrics including the Kullback–Leibler (KL), the Association Index (AI), and the Parsimony Score (PS). The KL measures divergence between the root state prior and posterior probability for each MCC tree. For this we used the program Matlab version 2011a [46] and a KL program written by Razavi [47]. The AI and PS test the null hypothesis that taxons, which contain a given trait such as a location, are no more likely to share that trait with adjoining taxa than by chance [48,49]. We used the program Bayesian Tip-Significance testing (BaTS) [50] to calculate the AI and the PS.

## Competing interests

The authors declare that they have no competing interests.

## Authors’ contributions

MS designed the study and wrote the manuscript. SV, CM, PR, MK, and AA reviewed the manuscript. YJM and JP contributed to the manuscript and reviewed the manuscript. INS contributed to the data analysis and reviewed the manuscript. All authors read and approved the final manuscript.

## Supplementary Material

Additional file 1 Map of Egyptian governorates with the number of HA sequences included in this study. Click here for file

Additional file 2 Map of Egyptian governorates with the number of NA sequences included in this study. Click here for file
