# Supplementary figures and images for: Phylogeography of influenza A H5N1 clade 2.2.1.1 in Egypt
Source: BMC Genomics. 2013 Dec 10;14:871. doi: 10.1186/1471-2164-14-871 (PMC3878885; doi:10.1186/1471-2164-14-871)

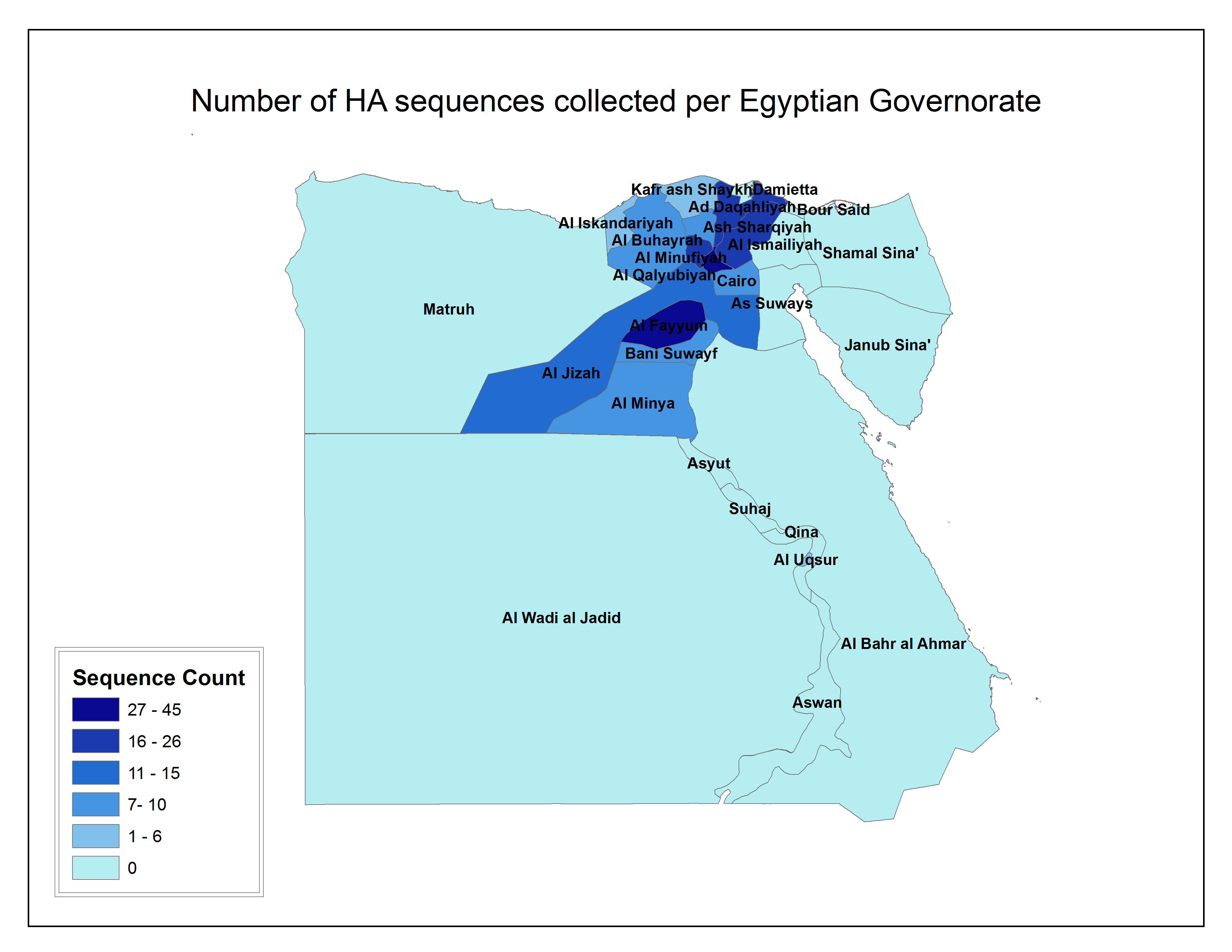

Supplement: Additional file 1 — Map of Egyptian governorates with the number of HA sequences included in this study. [file 1471-2164-14-871-S1.tiff]

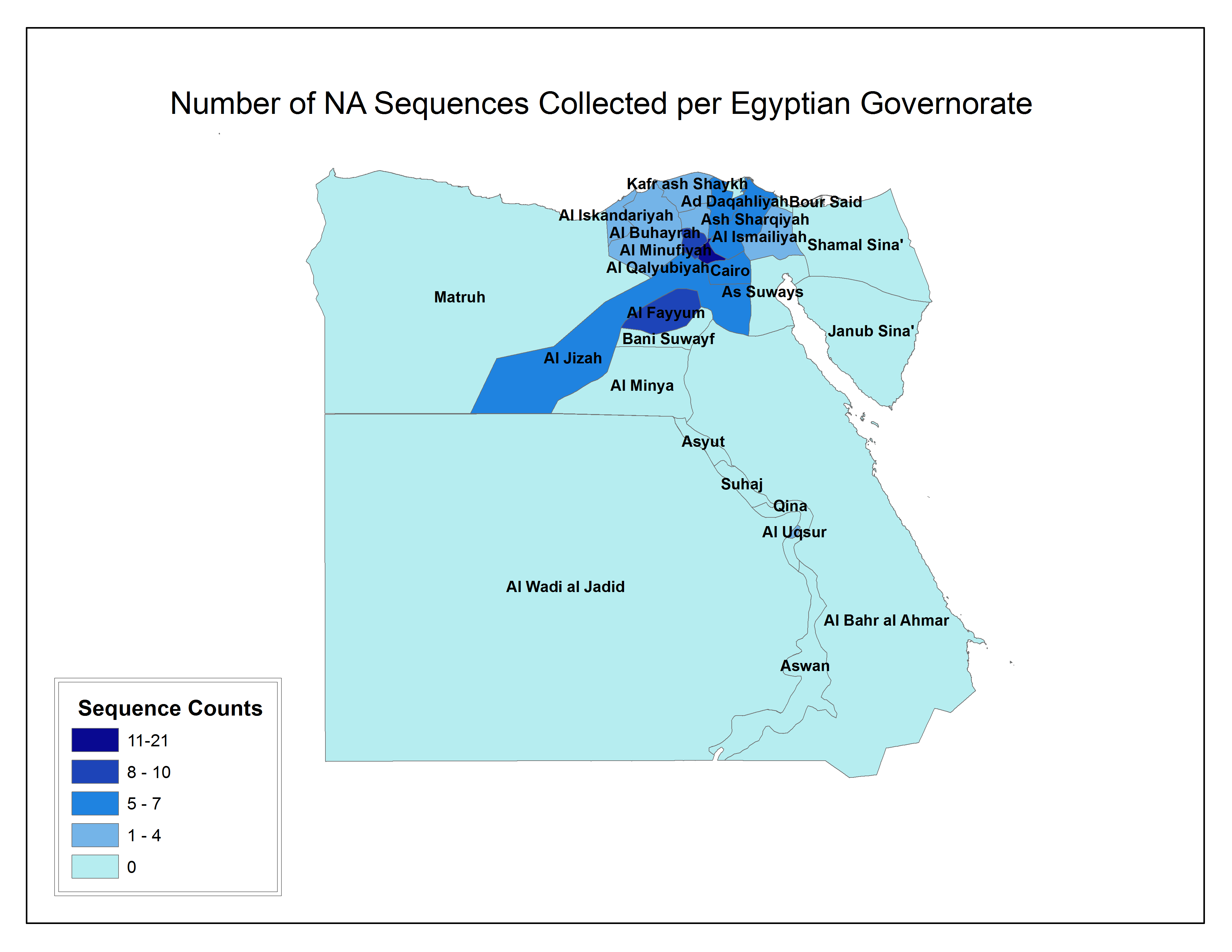

Supplement: Additional file 2 — Map of Egyptian governorates with the number of NA sequences included in this study. [file 1471-2164-14-871-S2.tiff]
